# Hematologic Inflammatory Indices Predict Mortality in Surgical Necrotizing Enterocolitis Among Premature Infants

**DOI:** 10.3390/children13020200

**Published:** 2026-01-31

**Authors:** Ahmet Dursun, İpek Kocaoğlu, Tülin Öztaş

**Affiliations:** 1 Department of Pediatric Surgery, Faculty of Medicine, Mugla Sıtkı Kocman University, 48000 Mugla, Turkey; 2 Department of Neonatology, Mugla Training and Research Hospital, 48000 Mugla, Turkey; ipekkc@gmail.com; 3 Department of Pediatric Surgery, University of Health Sciences, Gazi Yaşargil Training and Research Hospital, 21070 Diyarbakır, Turkey; tulinoztas@hotmail.com

**Keywords:** necrotizing enterocolitis, premature neonate, hematologic inflammatory indices, platelet to neutrophil ratio, mortality

## Abstract

**Highlights:**

**What are the main findings?**

- Preoperative and postoperative hematologic inflammatory indices (including NLR, PLR, SII, and PNR) were significantly associated with mortality in premature infants with surgically treated necrotizing enterocolitis. Postoperative inflammatory markers demonstrated stronger predictive value for mortality compared to preoperative values.

**What are the implications of the main findings?**

- Routine, easily obtainable hematologic indices may serve as practical prognostic tools to identify high-risk neonates with surgical necrotizing enterocolitis. Incorporating postoperative inflammatory markers into clinical decision-making may improve early risk stratification and guide postoperative monitoring and management.

**Abstract:**

**Background/Objectives**: Necrotizing enterocolitis (NEC) is one of the most devastating gastrointestinal emergencies in premature neonates, with particularly high mortality among those requiring surgical intervention. Early identification of high-risk patients remains challenging. This study aimed to evaluate the prognostic value of complete blood count-derived inflammatory indices for predicting mortality in premature infants undergoing surgery for NEC. **Methods**: A total of 74 premature neonates with Bell stage II or III NEC who underwent surgical treatment between 2018 and 2023 were retrospectively analyzed. Preoperative and postoperative hematologic inflammatory indices, including neutrophil-to-lymphocyte ratio (NLR), platelet-to-lymphocyte ratio (PLR), systemic immune-inflammation index (SII), and platelet-to-neutrophil ratio (PNR), were recorded. Receiver operating characteristic (ROC) curve analysis was used to assess predictive performance. Variables with *p* < 0.10 in univariate analysis were entered into multivariate logistic regression models. **Results**: Overall mortality was 35.1%. Non-survivors had significantly lower gestational age and birth weight and a higher prevalence of advanced disease. Preoperatively, NLR was higher and PNR was lower in non-survivors. Postoperatively, NLR and C-reactive protein levels increased, while PNR showed a marked decline in infants who died. ROC analysis identified postoperative PNR as the strongest predictor of mortality, followed by preoperative SII and postoperative NLR. Multivariate analysis demonstrated that lower gestational age, advanced disease stage, and reduced postoperative PNR were independently associated factors for mortality. **Conclusions**: Postoperative reduction in platelet-to-neutrophil ratio appears to be a practical, inexpensive, and easily obtainable biomarker for early risk stratification in surgically treated NEC. Incorporating routine hematologic inflammatory indices into postoperative monitoring may support timely identification of high-risk infants and guide individualized clinical management in neonatal intensive care units.

## 1. Introduction

Necrotizing enterocolitis (NEC) is a severe gastrointestinal emergency that occurs predominantly in very low birth weight (VLBW, <1500 g) and extremely premature neonates, characterized by high morbidity and mortality rates. The incidence of NEC among VLBW infants is reported to be 5–7% [1], and it represents one of the neonatal surgical emergencies with the highest mortality rates; approximately 20–30% of cases, particularly those requiring surgical intervention, result in death [2,3]. The pathogenesis of the disease is multifactorial, involving immaturity of the intestinal immune system, microbial dysbiosis, ischemia–reperfusion injury, and excessive activation of the inflammatory response [4].

Diagnosis of NEC relies on clinical, laboratory, and radiologic findings; however, these parameters are often nonspecific, particularly in the early stages of the disease. Therefore, there is an increasing need for reliable biomarkers capable of predicting disease progression and clinical outcome. In this context, complete blood count (CBC) parameters readily available and low-cost in routine clinical practice offer significant potential. The widespread availability, low cost, and rapid turnaround of CBC parameters provide a practical advantage for early risk assessment and monitoring of disease severity in NEC [5].

In recent years, several hematologic inflammatory indices derived from CBC parameters, namely the neutrophil-to-lymphocyte ratio (NLR), platelet-to-lymphocyte ratio (PLR), systemic immune-inflammation index (SII), and platelet-to-neutrophil ratio (PNR), have gained increasing attention as quantitative indicators of systemic inflammation and immune dysfunction. These indices have been reported to possess prognostic value in various neonatal inflammatory conditions such as sepsis, pneumonia, and NEC. However, most existing studies have focused on medically managed (non-surgical) NEC cases, and data specifically evaluating the association of these indices with mortality in premature infants requiring surgery are limited [6,7,8,9].

In surgically treated NEC, mortality rates remain considerably high, and long-term complications such as short bowel syndrome, malabsorption, and neurodevelopmental impairment are frequently observed [10,11,12]. Therefore, identifying hematologic markers capable of predicting early mortality risk in the surgical NEC population is of great clinical importance.

This study aimed to retrospectively evaluate the prognostic value of preoperative and postoperative hematologic inflammatory indices NLR, PLR, SII, and PNR as well as C-reactive protein (CRP), in predicting mortality among premature neonates undergoing surgery for NEC. We hypothesized that these easily accessible, inexpensive, and routinely measurable biomarkers could assist in the early identification of high-risk patients and help optimize postoperative management strategies.

## 2. Materials and Methods

This study was a retrospective, two-center observational investigation conducted in the Pediatric Surgery Departments of two tertiary training and research hospitals, with one center contributing the majority of cases. Clinical, laboratory, and surgical data of neonates who underwent surgical intervention for NEC between January 2018 and December 2023 were retrieved from electronic medical records and archived patient files. The study was conducted in accordance with the Declaration of Helsinki and approved by the institutional ethics committee (Approval No: 250055-03/2025).

All neonates diagnosed with NEC at Bell stage II or higher and who underwent surgical intervention were included in the study. The diagnosis of NEC was based on a combination of clinical findings (abdominal distension, bilious or bloody gastric content, rectal bleeding, abdominal tenderness, or discoloration) and radiologic features (pneumatosis intestinalis, pneumoperitoneum, and portal venous gas). The indication for surgery was determined by a multidisciplinary evaluation when intestinal perforation was radiologically demonstrated or when clinical deterioration persisted despite intensive medical therapy [13]. Surgery was performed urgently in cases of confirmed perforation; otherwise, it was undertaken when persistent clinical deterioration was observed despite intensive medical therapy (e.g., absence of stool passage, progressive abdominal distension, abdominal wall discoloration and bilious vomiting).

Patients with NEC secondary to congenital gastrointestinal malformations (such as Hirschsprung’s disease or intestinal atresia), those operated for meconium ileus or spontaneous intestinal perforation, those with known hematologic disorders that could affect hematologic profiles, and cases with incomplete laboratory data were excluded from the study.

The surgical approach was planned according to the patient’s clinical and radiologic findings. Based on intraoperative findings, bowel resection with primary anastomosis, stoma formation, or peritoneal drainage was performed. The choice of surgical method was determined jointly by the operating pediatric surgeon and the neonatal intensive care team through clinical consensus. Surgical indication and procedure type were recorded as baseline surgical variables and were considered as potential confounders in the statistical analysis.

Laboratory data included preoperative CBC parameters (white blood cell count, neutrophil, lymphocyte, hemoglobin, platelet) and CRP levels obtained at the time of surgical decision-making, and postoperative values obtained within the first 48 h after surgery (postoperative days 1–2). Hematologic inflammatory indices (NLR, PLR, SII, PNR) were calculated from these parameters. All hematologic counts (neutrophil, lymphocyte, and platelet) were derived from CBC results and expressed in the same unit (×10^9^/L) before calculation. In addition, CRP levels were evaluated as a biochemical indicator of the inflammatory response. Transfusion records were retrospectively reviewed, and when the timing information was available, complete blood count measurements used for index calculations were selected from samples obtained at least 24 h after platelet or red blood cell transfusion. In our clinical practice, platelet transfusion is routinely administered preoperatively when platelet count is <50,000/µL.

- NLR (Neutrophil-to-Lymphocyte Ratio) = Neutrophil/Lymphocyte
- PLR (Platelet-to-Lymphocyte Ratio) = Platelet/Lymphocyte
- SII (Systemic Immune-Inflammation Index) = (Platelet × Neutrophil)/Lymphocyte
- PNR (Platelet-to-Neutrophil Ratio) = Platelet/Neutrophil

Generative artificial intelligence tools were not used in the design of the study, data collection, analysis, or interpretation.

### Statistical Analysis

All statistical analyses were performed using IBM SPSS Statistics version 26.0. The normality of continuous variables was assessed using the Shapiro–Wilk test. Variables with normal distribution were expressed as mean ± standard deviation, while non-normally distributed variables were presented as median (interquartile range). Comparisons between groups were made using Student’s *t*-test or Mann–Whitney U test for continuous variables, and the Chi-square or Fisher’s exact test for categorical variables. Differences between preoperative and postoperative values were evaluated using the paired *t*-test or Wilcoxon signed-rank test when appropriate.

The predictive performance of hematologic indices for mortality was assessed by receiver operating characteristic (ROC) curve analysis and the Youden index. Variables with a *p* value < 0.10 in univariate analysis were included in the multivariate logistic regression model to identify independently associated factors for mortality. Because PNR, NLR, PLR, and SII are derived from overlapping complete blood count components, correlation analyses were performed to assess interrelationships and potential multicollinearity among these indices. To avoid collinearity, closely correlated indices were not entered simultaneously into the same multivariable model; instead, candidate indices were screened in univariable analyses and those showing significance were further evaluated in multivariable logistic regression together with clinically relevant covariates. In the final model, postoperative PNR was retained as the most informative marker. The goodness-of-fit of the model was evaluated using the Hosmer–Lemeshow test. A *p* value < 0.05 was considered statistically significant.

## 3. Results

### 3.1. Baseline Demographic and Clinical Characteristics

A total of 74 premature neonates who underwent surgical intervention for NEC were included in the study. The overall mortality rate was 35.1% (*n* = 26). When survivors (*n* = 48) and non-survivors (*n* = 26) were compared, non-survivors had significantly lower gestational age (26.4 ± 2.5 weeks vs. 28.1 ± 2.8 weeks; *p* = 0.009) and birth weight (870.2 ± 290.1 g vs. 1180.5 ± 430.5 g; *p* = 0.002). The 1st-minute Apgar score was lower in non-survivors (4.1 ± 1.7 vs. 5.2 ± 1.9; *p* = 0.012), as was the 5th-minute Apgar score (6.0 ± 1.6 vs. 6.9 ± 1.5; *p* = 0.021). The distribution of NEC stages differed significantly between the groups (*p* = 0.001), with a higher proportion of Stage III disease among non-survivors. No significant differences were observed regarding sex or maternal age (Table 1). Intraoperatively, necrotic bowel segments of varying extent were observed in most patients, and NEC totalis (diffuse intestinal necrosis) was identified exclusively in five infants in the non-survivor group. Mechanical ventilation was required in 35/48 (72.9%) survivors and 22/26 (84.6%) non-survivors; culture-proven sepsis was present in 12/48 (25.0%) survivors and 18/26 (69.2%) non-survivors.

### 3.2. Preoperative Laboratory Findings

In preoperative laboratory parameters, NLR was significantly higher in non-survivors compared with survivors (8.9 ± 8.1 vs. 4.9 ± 4.2; *p* = 0.007). Platelet counts were significantly lower in non-survivors (95.8 ± 55.3 vs. 136.8 ± 82.1 ×10^9^/L; *p* = 0.021), resulting in a significantly reduced PNR (16.2 ± 11.8 vs. 26.3 ± 23.1; *p* = 0.031). No significant differences were observed between the groups for PLR or CRP. Although mean SII values did not differ significantly between the groups, ROC-curve analysis demonstrated significant predictive performance for mortality.

### 3.3. Postoperative Laboratory Findings

In the postoperative period, inflammatory markers increased markedly in the non-survivor group. Postoperative NLR was significantly higher in non-survivors than in survivors (9.8 ± 7.9 vs. 5.9 ± 4.3; *p* = 0.008). Lymphocyte count (1.2 ± 1.0 vs. 1.8 ± 1.2 ×10^9^/L; *p* = 0.025) and platelet count (78.3 ± 43.1 vs. 123.5 ± 81.7 × 10^9^/L; *p* = 0.007) were significantly lower in non-survivors, leading to a pronounced postoperative decline in PNR (13.1 ± 10.2 vs. 25.1 ± 22.3; *p* = 0.004). Postoperative CRP levels were also significantly higher in non-survivors (94.5 ± 75.3 vs. 61.8 ± 55.2 mg/L; *p* = 0.016). In the postoperative period, SII values did not demonstrate significant prognostic differences.

### 3.4. Comparison of Preoperative and Postoperative Parameters in Survivors

Among survivors, comparison of preoperative and postoperative laboratory parameters showed significant postoperative decreases in lymphocyte count (2.2 ± 1.4 vs. 1.8 ± 1.2 ×10^9^/L; *p* = 0.008), platelet count (136.8 ± 82.1 vs. 123.5 ± 81.7 ×10^9^/L; *p* = 0.004), and hemoglobin levels (11.9 ± 3.5 vs. 10.5 ± 2.9 g/dL; *p* < 0.001). In contrast, postoperative CRP levels (48.1 ± 64.5 vs. 61.8 ± 55.2 mg/L; *p* = 0.029) and NLR values (4.9 ± 4.2 vs. 5.9 ± 4.3; *p* = 0.041) increased significantly.

### 3.5. ROC Curve Analysis of Hematologic Indices

**Figure 1:**
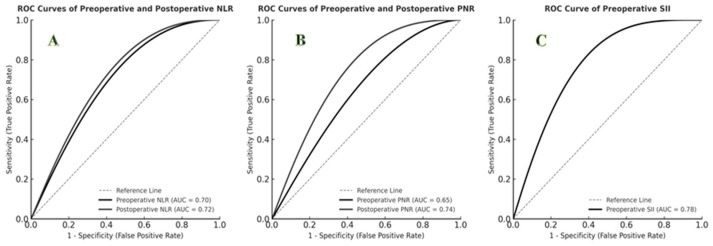
ROC curves for predicting mortality using hematologic indices. (**A**) ROC curves of preoperative and postoperative NLR (AUC = 0.70 and 0.72, respectively). (**B**) ROC curves of preoperative and postoperative PNR (AUC = 0.65 and 0.74, respectively). (**C**) ROC curve of preoperative SII (AUC = 0.78).

ROC curve analysis demonstrated that postoperative PNR had the highest predictive performance for mortality (AUC = 0.74; 95% CI: 0.62–0.86; *p* = 0.001; cut-off ≤ 15.0; sensitivity 77%; specificity 73%). Preoperative SII also showed strong predictive value (AUC = 0.78; cut-off = 1200; sensitivity 85%; specificity 70%). Both preoperative and postoperative NLR demonstrated significant predictive capacity (*p* < 0.01). The predictive performances of hematologic indices are summarized in Table 2, and ROC curves are shown in Figure 1.

### 3.6. Multivariate Logistic Regression Analysis for Mortality

Multivariate logistic regression analysis was performed to identify independently associated factors for mortality, including variables with *p* < 0.10 in univariate analysis and clinically relevant factors. The final model revealed that gestational age (OR: 0.68 per 1-week increase; 95% CI: 0.49–0.95; *p* = 0.026), advanced NEC stage (Stage III) (OR: 5.50; 95% CI: 1.59–18.98; *p* = 0.007), and postoperative PNR (OR: 0.94 per unit increase; 95% CI: 0.89–0.99; *p* = 0.010) were independently associated with mortality. The Nagelkerke R^2^ value of the model was 0.462, and the Hosmer–Lemeshow test indicated good model fit (*p* = 0.501). Univariate and multivariate logistic regression results are presented in Table 3. Correlation diagnostics confirmed substantial interdependence among the calculated indices; therefore, only one index was retained in the final multivariable model to minimize multicollinearity.

## 4. Discussion

In this two-center retrospective study focusing exclusively on surgically treated necrotizing enterocolitis, we demonstrated that postoperative PNR is independently associated with mortality and may serve as a clinically meaningful prognostic marker. This finding supports the clinical relevance of routinely available hematologic indices for postoperative risk stratification in surgically treated NEC. NEC remains one of the most devastating gastrointestinal emergencies in premature neonates, with mortality rates reaching 30–50% in surgically treated cases [14]. Despite advances in neonatal intensive care and surgical techniques, outcomes remain suboptimal, particularly among extremely preterm and very-low-birth-weight infants. Therefore, identifying reliable prognostic markers that can aid postoperative mortality risk estimation is important for improving clinical management. In this context, hematologic inflammatory indices such as PLR, NLR, SII, and PNR have attracted attention as accessible and cost-effective markers reflecting systemic inflammation and immune dysregulation.

The present study comprehensively identifies key demographic, clinical, and hematologic factors associated with mortality in premature neonates undergoing surgery for NEC. Gestational age (26.4 ± 2.5 weeks vs. 28.1 ± 2.8 weeks, *p* = 0.009) and birth weight (870.2 ± 290.1 g vs. 1180.5 ± 430.5 g, *p* = 0.002) were significantly lower in non-survivors. This finding supports the decisive role of prematurity-related organ immaturity, susceptibility to infection, and intestinal barrier dysfunction in mortality [15]. In addition, the association of low 1- and 5-min Apgar scores with mortality (*p* = 0.012 and *p* = 0.021, respectively) suggests that neonatal hypoxia may exacerbate the systemic consequences of NEC. The higher proportion of Bell stage 3 disease among non-survivors (57.7%) confirms the fundamental role of disease severity in determining prognosis. No significant differences were observed regarding sex or maternal age, suggesting limited impact of these factors on outcomes in surgical NEC. Collectively, these findings emphasize the need for close postoperative monitoring of preterm infants with advanced-stage NEC.

Preoperative hematologic findings reflect the inflammatory response and may help identify infants at higher risk of mortality in surgical NEC. The significantly higher NLR in non-survivors highlights the influence of neutrophil-mediated systemic inflammation and concomitant lymphopenia on poor outcomes. The decrease in platelet count may represent an early indicator of disseminated intravascular coagulation (DIC) and sepsis [16]. In addition, the significantly lower PNR in non-survivors (16.2 ± 11.8 vs. 26.3 ± 23.1; *p* = 0.031) suggests that this index, reflecting both inflammation and coagulopathy, may be a sensitive prognostic marker. In contrast, although previous studies have reported the prognostic value of CRP in predicting disease severity and the need for surgery [17], the absence of a significant difference in CRP levels between survivors and non-survivors in our cohort suggests limited utility of CRP for mortality discrimination in surgical NEC.

The Systemic Immune-Inflammation Index (SII) showed strong predictive ability in the preoperative period, with an AUC = 0.78. This result indicates that composite parameters reflecting the interaction among neutrophils, lymphocytes, and platelets may offer greater prognostic accuracy than individual indices alone. Although mean preoperative SII values did not differ significantly between survivors and non-survivors, ROC curve analysis demonstrated strong discriminatory power for mortality (AUC = 0.78, cut-off = 1200, sensitivity 85%, specificity 70%). This finding suggests that composite indices can capture immune-inflammatory interactions not reflected in single-parameter comparisons. It also underscores the potential role of SII as an integrative biomarker in surgical NEC. A previous study reported that elevated SII was superior to PLR in predicting surgical requirement, whereas low SII values were associated with increased mortality. In contrast, in our surgical NEC cohort, higher preoperative SII values predicted postoperative mortality. Together, these findings suggest that SII may be a phase-dependent biomarker with different prognostic implications according to the stage of the inflammatory process [9].

Although studies investigating the prognostic value of SII and NLR in NEC have been published, most have included medical or mixed NEC cases. By focusing exclusively on a surgical NEC cohort, our study provides a more homogeneous patient population. Moreover, the demonstration of an independent association between postoperative PNR and mortality constitutes the novel contribution of this study. While postoperative PNR remained independently associated with mortality, its ROC performance was lower than that of composite indices such as SII and NLR. This finding is consistent with previous reports suggesting that platelet-based ratios alone may be less sensitive to systemic inflammatory changes in advanced NEC [9,18].

The postoperative period represents a phase of maximal surgical stress and inflammatory response. The increase in postoperative NLR among non-survivors (9.8 ± 7.9 vs. 5.9 ± 4.3; *p* = 0.008) indicates persistence of systemic inflammation. In parallel, the decrease in lymphocyte count (1.2 ± 1.0 vs. 1.8 ± 1.2 × 10^9^/L; *p* = 0.025) may reflect immune suppression. Despite low platelet levels, the absence of severe thrombocytopenia (<50,000/µL) suggests that sepsis-related microthrombotic processes may progress rapidly without reaching terminal DIC stage. Therefore, monitoring platelet trends and considering abrupt declines as early warning signs may be clinically important.

One of the most notable findings of our study was the strong and independent association between postoperative PNR and mortality. PNR has been investigated as a marker of systemic inflammation and coagulopathy in various pediatric conditions, including kidney transplantation, neonatal sepsis, and pneumonia. However, studies examining this parameter in fatal surgical NEC remain scarce [19,20]. PNR reflects both the intensity of inflammatory response (neutrophilia) and the degree of coagulopathic alteration (thrombocytopenia), allowing a comprehensive assessment of postoperative prognosis. In ROC analysis, postoperative PNR demonstrated significant discriminatory power (AUC = 0.74; cut-off ≤ 15.0; sensitivity 77%; specificity 73%). Furthermore, multivariate logistic regression identified lower gestational age, advanced NEC stage, and lower postoperative PNR (OR = 0.94; *p* = 0.010) as independently associated factors for mortality, reinforcing the clinical relevance of PNR in the prognostic model.

These results suggest that postoperative PNR may serve as a practical biomarker for early assessment of mortality risk in surgical NEC. When interpreted alongside other hematologic indices such as NLR, PLR, and SII, it may provide additional prognostic information and support clinical decision-making. The prognostic performance of postoperative PNR likely reflects the pathophysiology of NEC, in which inflammation and platelet consumption are simultaneously activated.

Clinical Implications and Future Directions: Routine postoperative monitoring of NLR and PNR may facilitate early identification of high-risk infants and support individualized intensive-care management. In particular, incorporation of postoperative PNR into routine laboratory follow-up may provide a simple, low-cost approach for early risk stratification after surgery. These indices should be interpreted together with established clinical variables (gestational age, NEC stage, and overall clinical stability) to guide postoperative surveillance intensity. Future prospective multicenter studies with standardized sampling time points and detailed documentation of clinical confounders are needed to validate optimal cut-off values and determine whether index-guided monitoring strategies can improve outcomes.

Our study has several limitations. First, its retrospective design and inclusion of data from only two centers may limit the generalizability of the findings. Although we considered major clinical confounders (e.g., culture-proven sepsis, mechanical ventilation, vasoactive support, and transfusion exposure), the retrospective design and heterogeneous documentation limited comprehensive adjustment in multivariable models. This limitation is particularly relevant for platelet-based indices such as PNR; therefore, our results should be interpreted as prognostic associations rather than causal relationships. Hematologic parameters were evaluated at a single preoperative and postoperative time point, precluding assessment of their dynamic changes. Variations in surgical indications and timing decisions among clinicians may also represent a potential source of bias. Therefore, prospective large-scale multicenter studies incorporating serial measurements are needed to validate these results and confirm the prognostic utility of postoperative PNR.

## 5. Conclusions

Our study provides a comprehensive assessment of demographic and hematologic factors influencing mortality in premature neonates undergoing surgery for necrotizing enterocolitis. Gestational age, advanced disease stage, and postoperative platelet-to-neutrophil ratio emerged as independent and complementary predictors of mortality. Monitoring postoperative PNR may allow early identification of high-risk infants during the postoperative period. Integration of this simple, accessible, and low-cost parameter into routine clinical follow-up could serve as a practical tool for guiding individualized postoperative management strategies.

## Figures and Tables

**Table 1:** Comparison of demographic and perinatal characteristics between survivors and non-survivors.

Characteristic		Survivors (*n* = 48)	Non-Survivors (*n* = 26)	*p*-Value
Gestational age (weeks, mean ± SD)		28.1 ± 2.8	26.4 ± 2.5	0.009
Birth weight (g, mean ± SD)		1180.5 ± 430.5	870.2 ± 290.1	0.002
1-min APGAR score		5.2 ± 1.9	4.1 ± 1.7	0.012
5-min APGAR score		6.9 ± 1.5	6.0 ± 1.6	0.021
NEC stage III, n (%)		12 (25.0)	15 (57.7)	0.001
Sex (M/F)		27/21	15/11	NS *
Maternal age (years, mean ± SD)		29.4 ± 6.1	28.7 ± 5.9	NS *
**Surgical characteristics**				
Indication for surgery n (%)	Perforation	34 (70.8)	19 (73.0)	NS *
	Clinical deterioration (no perforation)	14 (29.2)	7 (17.0)	
Surgical procedure n (%)	Bowel resection + primary anastomosis	11 (23.0)	4 (15.4)	0.006
	Stoma formation	31 (64.5)	10 (38.5)	
	Peritoneal drainage	6 (12.5)	12 (46.1)	

* NS: not significant.

**Table 2:** Predictive performance of hematologic indices for mortality in surgical NEC.

Parameter	AUC (95% CI)	Cut-Off Value	Sensitivity (%)	Specificity (%)	*p*-Value
Postoperative PNR	0.74 (0.62–0.86)	≤15	77	73	0.001
Preoperative SII	0.78 (0.67–0.89)	≥1200	85	70	<0.001
Postoperative NLR	0.71 (0.58–0.83)	>7	69	68	0.004

**Table 3:** Univariate and multivariate logistic regression analysis for predictors of mortality in surgical NEC.

Variable	Univariate OR (95% CI)	*p*-Value	Multivariate OR (95% CI)	*p*-Value
Gestational age (per week ↑ *)	0.70 (0.52–0.94)	0.018	0.68 (0.49–0.95)	0.026
NEC stage III	5.10 (1.70–15.40)	0.005	5.50 (1.59–18.98)	0.007
Postoperative PNR (per unit ↑)	0.95 (0.91–0.99)	0.012	0.94 (0.89–0.99)	0.010
Birth weight (per 100 g ↓)	0.96 (0.90–1.02)	0.142	—	—
Preoperative NLR	1.09 (0.99–1.21)	0.074	—	—

* Arrows indicate the direction of change (↑ increase, ↓ decrease).

## Data Availability

The data presented in this study are available from the corresponding author upon reasonable request. The data are not publicly available due to privacy and ethical restrictions.
